# Surgical recurrence of solitary fibrous tumor of the pleura treated with microwave (MW) thermoablation: A case report

**DOI:** 10.1111/1759-7714.13263

**Published:** 2019-12-26

**Authors:** Francesco Fiore, Vincenzo Stoia, Francesco Somma

**Affiliations:** ^1^ Interventional Radiology Unit National Cancer Institute “IRCCS Fondazione Pascale” Napoli Italy

**Keywords:** Microwave, pleura, solitary fibrous tumor, thermoablation

## Abstract

Solitary fibrous tumor (SFT) of the pleura is a rare neoplasm which is challenging for clinicians to treat and radiologists to diagnose. Herein, we report a case of recurrence of SFT of the pleura in a 77‐year‐old patient which was diagnosed and surgically treated on the first occasion in 2005. The patient had a recurrence in 2016 which was treated and then six months later, he again experienced chest pain and a further local recurrence was found. Taking into consideration the age and comorbidities of the patient, CT‐guided percutaneous microwave‐thermal ablation was preferrable to surgery and a safe and highly effective local ablative technique with few side‐effects.

## Key points

Microwave thermoablation is an effective and safe technique for pleural solitary fibrous tumor.Microwave thermoablation helps reduce tumor‐related pain in patients affected by pleural solitary fibrous tumor.

## Introduction

Solitary fibrous tumor (SFT) of the pleura represents less than 5% of all pleural neoplasms. Effusions are frequently large, occupying 50% or more of the hemithorax and obscuring the pleural tumor.^1^ The tumor generally spreads by local invasion throughout the pleural cavity to the chest wall, axilla and supraclavicular area. Extra‐thoracic localization (meninges, nose, oral cavity, pharynx, thyroid, breast, kidney, spinal cord) is also possible but exceedingly rare. The diaphragm, as well as the surface of the peritoneum, may also be involved. Distant metastatic disease is unusual, involving liver, bone, brain, adrenal gland, kidney, pancreas, thyroid, spleen, skin and lymph nodes.^1^ Hilar and mediastinal lymph node involvement occurs in less than 50% of patients. Extrathoracic lymph node involvement is very rare. Despite being clinically similar to asbestos‐related neoplasms, this tumor has a distinct pathological entity,^2^ and immunoprofile evaluation has confirmed its mesenchymal origin. The tumor arises from the pleura, is either visceral (80%) or parietal (20%) and may undergo malignant transformation.^3^ Thermoablation techniques such as cryoablation, radiofrequency ablation (RFA), microwave ablation (MWA), laser ablation, and high intensity focused ultrasound (HIFU), have an established role in the treatment of localized tumors, especially in cases of well differentiated and/or radio‐resistant neoplasms. To our knowledge, this is the first case of surgical recurrence of solitary fibrous tumor of the pleura successfully treated with microwave thermoablation.

## Case report

In September 2005, a 65‐year‐old patient attended the clinic complaining of exertional dyspnea. His medical history was unremarkable and laboratory tests were normal. Chest X‐ray revealed an enormous space‐occupying lesion in the left hemithorax which was causing left lung collapse and right‐sided mediastinal shift. Multi‐detector computed tomography (MDCT) confirmed these features, but the relationship to the parietal pleura and mediastinal structures was unclear. Intravenous contrast administration with multiphase acquisition showed high vascularity of the tumor, with no lymphoadenopathy or compression of the left pulmonary artery. Surgical thoracotomy showed an encapsulated tumor, occupying the left pleural cavity and compressing the lung without invasion of mediastinal structures. The tumor was surgically removed en bloc and the left lung immediately re‐expanded. The tumor was enormous measuring 30 × 19 cm and weighed 4050 g.^4^ Microscopy showed fibrotic tissue with large areas of hyalinization, but there was no evidence of malignancy. Immunohistochemical analyses were positive for CD 34, and negative for vimentin and cytokeratin. The histologic diagnosis was SFT of the pleura Stage 0 according to the De Perrot classification.^5^, ^6^

**Figure 1:**
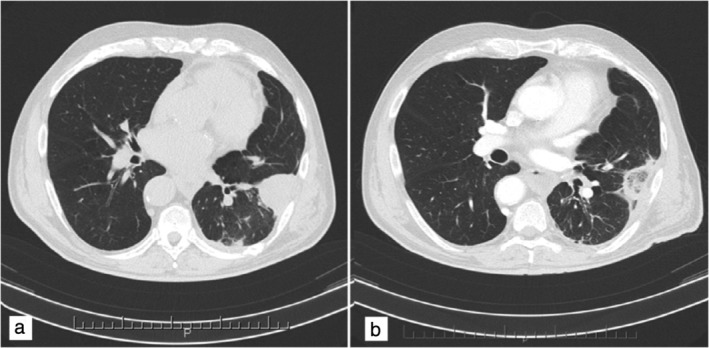
Solitary fibrous tumor of the pleura. Multi‐detector computed tomography (MDCT) revealed a focal pleural thickening at the lower left lobe (**a**) before and (**b**) three months after microwave thermal ablation. Fibrotic retraction of the lesion showed dyshomogeneous aspect due to multiple necrotic areas and cavitation within the lesion.

**Figure 2:**
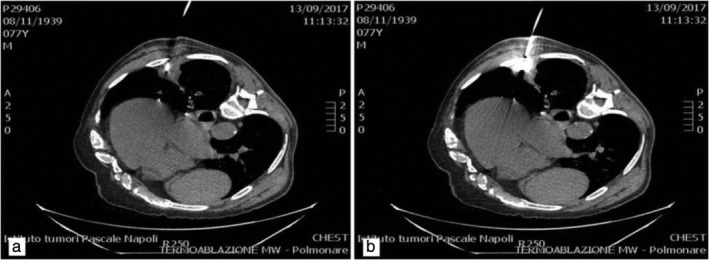
(**a**) Solitary fibrous tumor of the pleura. (**b**) Under multi‐detector computed tomography (MDCT) MW guidance, a needle was placed in the very center of the lesion.

**Figure 3:**
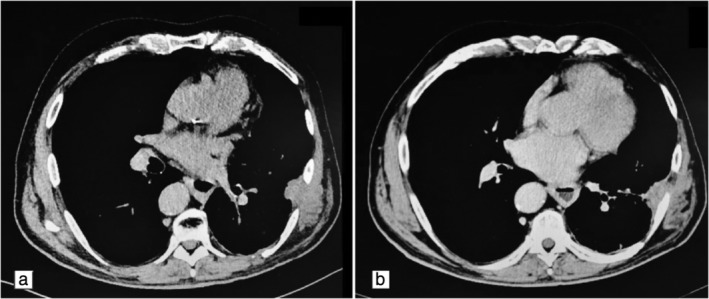
(**a**) Solitary fibrous tumor of the pleura. (**b**) At 36 months MDCT follow‐up showed further fibrous retraction of the lesion.

After a period of 11 years, in April 2016, the patient returned to the clinic for a routine chest X‐ray which showed a small rounded area of high attenuation, confirmed by multiphase MDCT, and the mass was surgically removed a few weeks later. In October 2016 a new mass of 40 x 36 mm was found in a different pleural location (Fig 1a) during a MDCT scan control (64/row, Optima 660 GE Healthcare USA MDCT; scans parameters 120 KVp 100–470 mAs (NI16.36), 2.5 mm slice table speed 0.984/1 mm/rotation). In this case, the lesion was more focused and a biopsy confirmed the diagnosis of recurrence of SFT of the pleura. On this occasion, MDCT‐guided MWA was considered to be the more appropriate ablation technique because the lesion size was 40 x 36 mm, the mass was located close to the pleura but distant from cardiovascular structures, and ablation time was reduced compared to other locoregional treatment. Before the procedure, the patient was adequately informed and his consent to the procedure was obtained. The procedure was performed under MDCT guidance with a low‐dose scanning protocol in order to reduce the radiation dose administered to the patient as much as possible.After local anesthetic cutaneous and subcutaneous administration, the procedure was managed using analgosedation with the benzodiazepines propofol and fentanyl. The microwave (MW) angiodynamics Solero produces 0 W to 140 W of power at a frequency of 915–2450 MHz. A MW 14 G needle‐antenna using the water circulation cooling system was placed in the very center of the lesion and wattage was administered. Lesion ablation treatment was performed with about 140 W for two minutes and 100 W for the following four minutes (Fig 2). At the end of the treatment neither pneumothorax nor pleural effusion was observed. Three months post‐treatment, MDCT control showed several necrotic areas within the lesion, and no enhancement after contrast medium administration (Fig 1b). The patient was dismissed the day after the procedure in very good condition. Follow‐up MDCT (Fig 3) was performed at 36 months and showed a little fibrotic area without any sign of pathological enhancement due to local recurrence, and the patient was in good health. Our assessment, therefore, is that MW thermoablation is a safe and highly effective local ablative technique with few side‐effects.

## Discussion

Microwave ablation (MWA) is a widely used ablation technique based on inducing necrosis through high temperatures. MWA systems generate an ellipsoidal microwave field around a needle‐like applicator that is introduced into the tissue. Since water molecules have a positively and a negatively charged pole, they tend to align with the electromagnetic waves. The oscillation of the electromagnetic wave therefore causes a rapid flip motion of the water molecules which results in heating of the adjacent tissue through the mechanism of dielectric hysteresis. If the MW frequency perfectly matches the molecule‐specific resonance frequency of the water molecules, all the energy will be turned into heat, but the penetrability into the tissue will be low. The frequencies used by the current MW manufacturers (915 MHz and 2450 MHz) only partially match the resonance frequency of the water molecules, thus assuring efficient energy conversion into heat with satisfactory tissue penetrability.^7^, ^8^, ^9^ MWA generally produces larger necrotic thermocoagulation that is less dependent on tissue properties. Tissue changes caused by ablation, such as carbonization and desiccation, also increase tissue resistivity, thus further hindering the expansion of the ablation area.^10^, ^11^ The antenna insertion technique is very similar to CT‐guided biopsies. Immediately prior to the intervention, an unenhanced CT scan of the chest is performed in order to plan the best puncture approach. The puncture site must be properly disinfected and isolated with sterile drapes before local anesthesia is applied to the skin and pleura. Following a small skin incision using a scalpel, the antenna is inserted under breath‐hold as close as possible to the center of the tumor. Single CT is then used to verify the position of the antenna and to correct it, if necessary.^12^, ^13^, ^14^ The success of the ablation depends mainly on the size of the ablation margin which depends on the size of the tumor, size of the ablation zone and position of the antenna relative to the lesion.

In conclusion, MW thermoablation is safe and highly effective for the local ablative treatment of lung lesions and particularly in cases of SFT of the pleura recurrence after surgery. Moreover, this technique avoids long‐term hospitalization and all the risks associated with surgery.

## Disclosure

The author declares there is no conflict of interest.
